# DSF/Cu induces antitumor effect against diffuse large B-cell lymphoma through suppressing NF-κB/BCL6 pathways

**DOI:** 10.1186/s12935-022-02661-4

**Published:** 2022-07-26

**Authors:** Yunying Zhu, Chenshuang Lei, Qian Jiang, Qinhua Yu, Liannv Qiu

**Affiliations:** 1 Department of Clinical Laboratory, College of Medical Technology, Zhejiang Chinese Medical University, Hangzhou, 310014 Zhejiang China; 2 Laboratory Medicine Center, Department of Clinical Laboratory, Zhejiang Provincial People’s Hospital (Affiliated People’s Hospital, Hangzhou Medical College), 158 Shangtang Road, Hangzhou, 310014 Zhejiang China

**Keywords:** Diffuse large B-cell lymphoma, Disulfiram, Apoptosis, B-cell lymphoma 6, Cell cycle arrest, Nuclear factor kappa beta

## Abstract

**Background:**

The B-cell lymphoma 6 (*BCL6*) oncogene is required for the survival of diffuse large B-cell lymphoma (DLBCL), which is incurable using conventional chemotherapy. Thus, it is imperative to improve the survival of patients with DLBCL. Disulfide (DSF) has been shown to have anticancer effects, but its effect on DLBCL remains unclear.

**Methods:**

Four DLBCL cell lines (OCI-LY1, OCI-LY7, OCI-LY10 and U2932) and primary DLBCL cells from eight newly diagnosed DLBCL patients were pretreated with DSF alone or in combination with Cu. Cell morphology was observed under microscope. Flow cytometry was performed to evaluate the cell apoptosis, cell cycle, the mitochondrial membrane potential and the intracellular accumulation of reactive oxygen species (ROS). The protein expression was respectively measured by flow cytometry and western blotting.

**Results:**

DSF or DSF/Cu exhibited a marked inhibitory effect on the growth of DLBCL cells, accompanied by cell cycle arrest at the G0/G1 phase. Meanwhile, DSF or DSF/Cu significantly induced DLBCL cells apoptosis. Further study revealed that DSF or DSF/Cu promoted apoptosis by inhibiting NF-κB signaling pathway. Interestingly, DSF/Cu significantly reduced BCL6 and AIP levels. In addition, DSF significantly up-regulate p53 protein in OCI-LY7 and OCI-LY10 while down-regulate p53 protein in OCI-LY1 and U2932.

**Conclusion:**

These results provided evidence for the anti-lymphoma effects of DSF on DLBCL and suggested that DSF has therapeutic potential to DLBCL.

**Supplementary Information:**

The online version contains supplementary material available at 10.1186/s12935-022-02661-4.

## Background

Diffuse large B-cell lymphoma (DLBCL) is an aggressive and rapidly progressing molecularly-heterogeneous disease which accounts for 40% of new non-Hodgkin lymphoma (NHL) [1–3]. Gene expression profiles have identified subtypes of DLBCL [activated B-cell-like (ABC), germinal-center B-cell-like (GCB), and unclassified] according to the cell of origin, which are associated with differential responses to chemotherapy and targeted agents. DLBCL subtypes have significant differences in clinical outcome, with the ABC-DLBCL subtype being associated with a poor outcome [4, 5]. Currently, the main treatment for DLBCL is using first-line management consisting of rituximab, cyclophosphamide, doxorubicin, vincristine and prednisone (R-CHOP) [6, 7]. Nevertheless, a significant proportion of patients with DLBCL will relapse or develop refractory disease, and most patients with relapsed or refractory DLBCL will succumb to the disease [8, 9]. Therefore, new chemotherapeutic prophylaxis and/or chemotherapeutic agents are urgently needed in order to improve poor outcomes.

The B-cell lymphoma 6 (*BCL6*) gene, initially found in B-cell lymphoma as a proto-oncogene, drives the malignant phenotype by inhibiting gene transcription and DNA damage checkpoints and blocking B-cell terminal differentiation. *BCL6* is a transcriptional suppressor of the pox virus and zinc finger (POZ)/broad-complex, tramtrack and bric a brac (BTB) zinc-finger protein family, acting as a key regulator for GC development and further differentiation. Most B-cell lymphomas occurring in germinal center (GC) B cells require continuous expression of BCL6 for survival [10–14]. The *MEF2B* gene mutation results in enhanced transcriptional activity, increased BCL6 expression, drives lymphomagenesis in mouse model, led to GC enlargement and lymphoma development [15]. Additionally, the reduction or loss of FBXO11 led to an increased number of GC B cells and higher levels of BCL6 protein in GC B cells which promote lymphoproliferative disorder [16]. Aryl Hydrocarbon Receptor Interacting Protein (AIP), the molecular chaperone of heat shock protein 90, regulates the growth and differentiation of B cells and is highly expressed in DLBCL. AIP protects BCL6 from FBXO11-mediated proteasome degradation by binding to ubiquitin ligase UCHL1 [17]. Thus, BCL6 could affect DLBCL by modulating B-cell activation, differentiation, cell cycle arrest and apoptosis [18, 19]. In addition, BCL6 is also involved in the development of CD4^+^ T-follicular helper cells that play a critical role during the generation of the GC [20, 21]. Targeted inhibition of BCL6 is a potential therapeutic strategy for DLBCL, and BCL6 may be a good therapeutic target for DLBCL therapies and combinatorial regimens.

Disulfiram (DSF) is a clinical anti-alcohol drug with a good safety record at fDA-recommended doses [22]. DSF has demonstrated antitumor effect in some tumors, including leukemia, melanoma, thymic mesothelioma and breast cancer [23–25]. DSF can also improve the antitumor effect of some chemotherapy drugs and radiation, as well as simultaneously protect kidney, intestinal, and bone marrow from cytotoxic drugs [26–29]. When DSF was used in combination with copper, it showed higher cytotoxicity [26]. DSF/Cu can target breast cancer stem cells through ROS-nuclear factor kappa beta (NF-κB) signaling pathways [28]. In acute lymphocytic leukemia, DSF/Cu can reduce mitochondrial membrane potential and induce apoptosis through down-regulating the anti-apoptotic proteins BCL2 and BCL-XL [30]. DSF/Cu could induce apoptosis by the alteration of the ROS levels and inhibiting both aldehyde dehydrogenase and NF-κB activities [28, 31]. DSF inhibits the activity of P-glycoprotein which mediates drug sensitivity [32]. Nevertheless, the detailed antitumor mechanisms of DSF on DLBCL remain to be fully elucidated.

The present study aimed to explore the role and potential antitumor mechanism of DSF/Cu on DLBCL. Our results demonstrated that DSF/Cu inhibited cells growth and significantly induced apoptosis in DLBCL cells. Further research revealed that the mechanism involved regulation of the mitochondrial transmembrane potential, the intracellular ROS levels, and NF-κB signaling pathway. Most importantly, DSF or DSF/Cu significantly reduced BCL6 levels. These results indicated that DSF may be a new BCL6 inhibitor with therapeutic potential for DLBCL.

## Methods

### Cell culture and treatment

The human DLBCL-derived cell lines OCI-LY1, OCI-LY7, OCI-LY10 and U2932 cell lines were kindly donated by Professor Chen Zeshi at the Kunming Institute of Zoology, Chinese Academy of Sciences. The primary DLBCL cells were derived from 8 newly diagnosed DLBCL patients (female 4 and male 4, aged 45–85 years) (Table 1). Zhejiang Provincial People’s Hospital Ethics Board approved this study and informed consent for the sample analysis. Primary CD19^+^ B cells were isolated immediately using Ficoll gradient centrifugation according to the manufacturer’s instructions. After 1 h of incubation at 37 °C in 5% CO_2_, adhesive mononuclear cells were removed. CD19^+^ B cells was confirmed to be > 90% by flow cytometry. The GCB-DLBCL cell lines, OCI-LY1 and OCI-LY7, were cultured in complete Iscove’s modified Dulbecco’s medium (IMDM; Hyclone; Cytiva) while ABC-DLBCL cell lines OCI-LY10, U2932 and primary DLBCL cells were cultured in Roswell Park Memorial Institute (RPMI)-1640 medium (Hyclone; Cytiva). All cells were cultured in medium supplemented with 10% fetal bovine serum (FBS; Gibco; Thermo Fisher Scientific, Inc.), penicillin (100 U/mL) and streptomycin (100 µg/mL) (Hyclone; Cytiva) in an incubator humidified with 5% CO_2_ at 37 °C.

**Table 1:** Biological characteristics of primary DLBCL patients

Patient	Age	Sex	Type	Disease status	Previous treatment	TP53 status	BCL6	BCL2	c-MYC	CD20	CD10	Ki67 (%)	MUM-1
1	63	M	ABC-DLBCL	D	NO	mut	NS	+	NS	+	−	40–50	−
2	45	M	GCB-DLBCL	D	NO	mut	+	−/+	+	+	+	95	+
3	55	F	ABC-DLBCL	D	NO	mut	−	−	−	+	−	37.2	−
4	53	F	ABC-DLBCL	D	NO	mut	−	+	−	+	−	60	−
5	53	F	ABC-DLBCL	D	NO	del/wt	−	+	−	+	−	50	+
6	68	M	ABC-DLBCL	D	NO	mut	+	+	−	+	−	60	+
7	85	F	GCB-DLBCL	D	NO	wt	+	+	−	+	−	40	−
8	57	M	GCB-DLBCL	D	NO	del/wt	−	−	−	+	−	70	−

D, diagnosis; wt, wild type; del, deletion; mut, mutation

### Reagents

A 500 mM DSF (Sigma-Aldrich; Merck KGaA) stock solution was dissolved in high-quality anhydrous dimethyl sulfoxide (DMSO) and stored in the dark at − 20 °C. A stock solution of copper gluconate (Sigma-Aldrich; Merck KGaA) at a concentration of 500 μM was prepared by dissolving the compound in ddH_2_O. A 500 mM *N*-acetyl-l-cysteine (NAC; Sigma-Aldrich; Merck KGaA) stock solution was dissolved in high-quality anhydrous DMSO and stored in the dark at − 20 °C. Antibodies for western blot analysis were as follows: caspase 3 (cell signaling technology), β-actin (ProteinTech), AIP (cell signaling technology), p53 (cell signaling technology), BCL6 (cell signaling technology); cleaved (c)-caspase 3 (Abcam), BCL2 (Abcam), BCL-XL (Abcam), IκB (Abcam), phosphorylated (p)-IκB (Abcam), NF-κB p65 (Abcam) and BAX (HuaBio). Antibodies for flow cytometry were as follows: Alexa Fluor^®^ 647-conjugated BCL2 (clone Bcl-2/100; BD Pharmingen™; BD Biosciences), Alexa Fluor^®^ 488-conjugated BAX (clone 2D2; BioLegend.), PE-Cy™7-conjugated BCL6 (clone K112-91; BD Biosciences) and PE-conjugated BCL-XL (clone 7B2.5; Invitrogen; Thermo Fisher Scientific, Inc.). The Cell Counting Kit-8 (CCK-8) was purchased from Genview. JC-1 Mitochondrial Membrane Potential Detection Kit (C2006) was purchased from Beyotime Institute of Biotechnology. Dihydrorhodamine (DHR) 123 (D1054) was purchased from Sigma-Aldrich; Merck KGaA.

### CCK-8 cytotoxicity assay

The suppressive effect of DSF, copper gluconate, and their combination (DSF/Cu) on metabolic activity was assessed using the CCK-8 assay. Briefly, OCI-LY1, OCI-LY7, OCI-LY10 and U2932 cells (1.5 $\times$ 10^4^ cells/well) were placed into 96-well plates and incubated at 37 °C for 24, 48 and 72 h. The copper gluconate concentration used in the present study was similar to the physiological copper concentration [33]. DMSO was used as a control. Subsequently, 10 μL of CCK-8 solution was added to each well and incubated at 37 °C for 4 h before the absorbance was measured. The absorbance was measured at 460 nm using a microplate reader (Tecan Infinite M200; Tecan Group Ltd.). The 50% cell growth inhibitory concentration (IC_50_) was defined as the concentration that inhibited cell growth by 50% compared with untreated control. Three replicates per treatment were evaluated and each experiment was repeated three times.

### Hoechst 33258 and Wright staining

For Hoechst 33258 staining, DLBCL cells (5 $\times$ 10^5^ cells) were fixed with fixative methanol:glacial acetic acid (3:1) for 5 min at 4 °C and stained with 10 μg/mL of Hoechst 33258 (Applygen Technologies Inc.) for 10 min. Morphological changes associated with apoptosis were observed using fluorescence microscopy (Nikon Y-THS; Nikon Corporation) with 350- to 370-nm excitation wavelengths and emission detection at 465 nm.

Concurrently, the cells were stained with Wright (BaSO) and morphological changes were observed under a microscope (TS100-F: Nikon Corporation) following the designated treatment.

### Analysis of cell apoptosis

The apoptosis assessment was evaluated via Annexin V/PI assay (Beckman Coulter, Inc.) using a NAVIOS flow cytometer (Beckman Coulter, Inc.) according to the manufacturer’s protocol. Annexin V^+^ apoptotic cells include Annexin V^+^PI^−^ early apoptotic cells and Annexin V^+^ PI^+^ late apoptotic cells.

### Cell cycle analysis

The cell cycle analysis was performed using a DNA Prep™ reagent Kit (Beckman Coulter, Inc.) by NAVIOS flow cytometry (Beckman Coulter, Inc.) according to the manufacturer’s protocol. The fractions of the cell population in the G1/G0 (G01), S, and G2/M phases were quantified via using the Wincycle32 software (Beckman Coulter, Inc.). The sub-G1 fraction was determined from the total event count.

### Mitochondrial transmembrane potential (ΔΨm) analysis

The mitochondrial depolarization that occurred during apoptosis was measured and analyzed by flow cytometry using a JC-1 staining. The cells (5 $\times$ 10^5^ cells/mL) were mixed with JC-1 probe (Beyotime Institute of Biotechnology) at a working concentration of 10 μg/mL for 20 min at 37 °C.

### Intracellular ROS detection

DHR 123 was used to detect intracellular ROS. The cells (5 $\times$ 10^5^ cells/mL) were exposed to 10 μM DHR for 1 h at 37 °C. Finally, the mean fluorescence intensity of the cells was detected using flow cytometry. The data was analyzed with 10.6.2 FlowJo software.

### Western blot analysis

Cells were harvested, washed and suspended in RIPA lysis buffer (Wuhan Servicebio Technology Co. Ltd.). Following 6000*g* centrifugation for 10 min at 4 °C, the supernatant was collected. The total protein in each sample was quantified using a bicinchoninic acid (BCA) kit (Wuhan Servicebio Technology Co. Ltd.). Equal amounts of proteins (10 µg/lane) were electrophoretically separated via 12% SDS-PAGE and transferred to a PVDF membrane (EMD Millipore). The membrane was blocked for 1 h in 5% skimmed milk dissolved in 0.1% of Tween-20 used for TBST at 4 °C and then incubated with antibodies directed against, human caspase 3 (1:1000), c-caspase 3 (1:1000), IκB (1:1000), p-IκB (1:1000), NF-κB p65 (1:1000), BCL2 (1:1000), BCL-XL (1:1000), BAX (1:1000), AIP (1:1000), p53 (1:1000), and β-actin (1:1000) at 4 °C overnight, followed by incubation with goat anti-rabbit IgG H&L (HRP) (1:1000; A0216, Beyotime Institute of Biotechnology) or goat anti-rat IgG H&L (HRP) (1:1,000; A0208, Beyotime Institute of Biotechnology) at room temperature for 1 h. The protein signals were detected using Super electrochemiluminescence (ECL) Detection Reagent (Yeasen Biotechnology Co. Ltd) by 5.2 Image Lab software (Bio-Rad Laboratories, Inc.). The bands on the membrane were quantified by normalization to β-actin.

### Statistical analysis

All experiments were performed independently three times. Data are presented as the mean ± standard deviation (SD) of three independent experiments. The difference between the drug-treated group and the control group was analyzed using unpaired Student’s t-test with 19.0 SPSS software (IBM Corp.). *P* < 0.05 was considered to indicate a statistically significant difference.

## Results

### DSF/Cu inhibits the proliferative viability of DLBCL cells

**Fig. 1:**
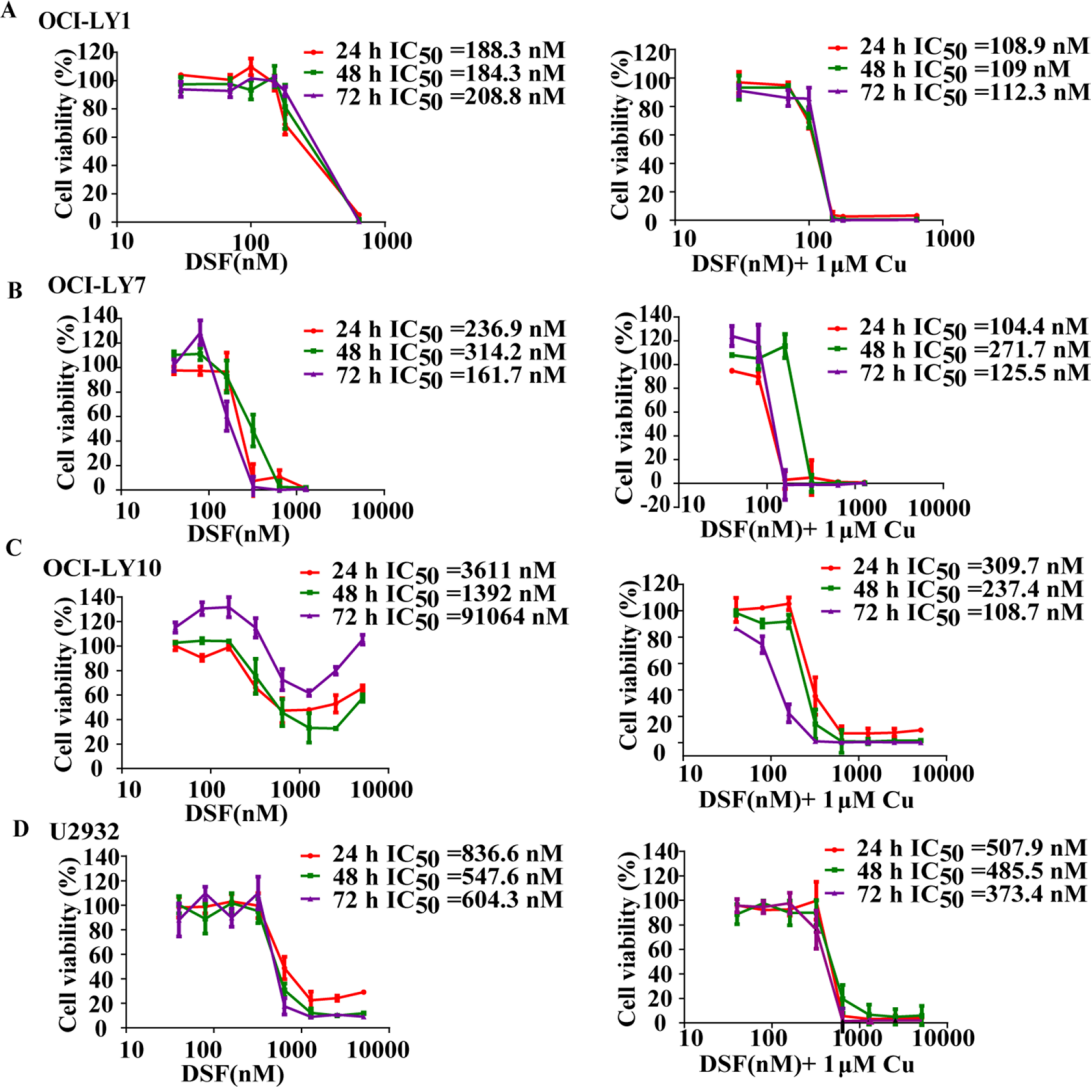
DSF/Cu inhibits the proliferative viability of DLBCL cells. CCK-8 assays detected the proliferation activity of OC-LY1 (**A**), OC-LY7 (**B**), OC-LY10 (**C**) and U2932 (**D**) cells treated with different concentrations of DSF or DSF/Cu for 24 h, 48 h or 72 h

To determine the inhibitory effect of DSF or DSF/Cu on DLBCL cells, cell viability was first evaluated using the CCK-8 assay in four DLBCL cell lines (OCI-LY1, OCI-LY7, OCI-LY10 and U2932). The CCK-8 assay revealed that DSF or DSF/Cu inhibited cell proliferation significantly in a time or dose-dependent manner, and the inhibitory effect was more significant following DSF/Cu treatment than after DSF treatment alone in all four cell lines (Fig. 1A–D). Copper gluconate alone at 1 μM did not alter the cell viability.

The IC50 of DSF or DSF/Cu in the four DLBCL cell lines was examined, and it was revealed that the four DLBCL cells had different sensitivities to DSF or DSF/Cu, suggesting that the cytotoxic effect of DSF is enhanced in the presence of copper (Fig. 1A–D). According to the CCK-8 results, the concentration of DSF in the next experiments was based on the IC50 of 24 h (OCI-LY1: 108.9 nM, OCI-LY7: 104.4 nM, OCI-LY10: 309.7 nM, U2932: 507.9 nM, Cu: 1 μM). Under microscopy, it was observed that DSF or DSF/Cu-treated DLBCL cells no longer gathered into clusters, and the number of living cells gradually decreased (Additional file 1: Fig. S1). These results demonstrated that DSF or DSF/Cu exhibited marked cytotoxicity toward DLBCL cells.

### DSF/Cu causes G0/G1 cell cycle arrests in DLBCL cells

**Fig. 2:**
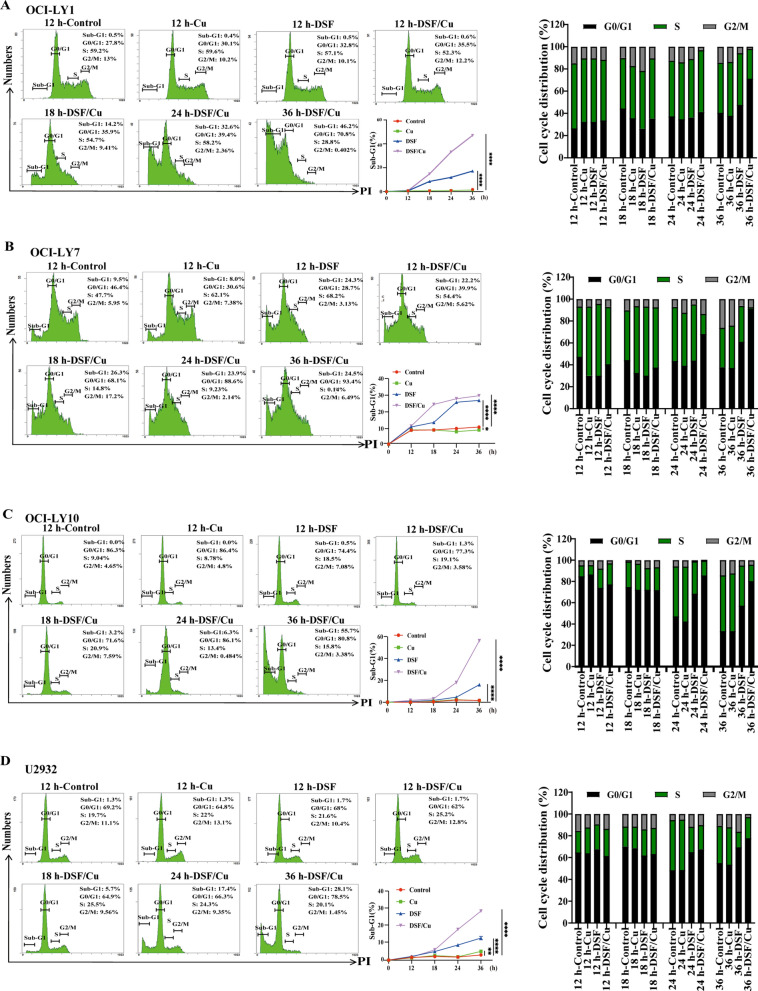
DSF/Cu causes G0/G1 cell cycle arrests in DLBCL cells. The effects of different durations of 1 μM Cu, DSF (OCI-LY1 108.9 nM; OCI-LY7 104.4 nM; OCI-LY10 309.7 nM; U2932 507.9 nM), and DSF/Cu on Sub G1 and the cell cycle of OCI-LY1 (**A**), OCI-LY7 (**B**), OCI-LY10 (**C**) and U2932 (**D**) cells

Uncontrolled cell proliferation is the hallmark of cancer and tumor cells are directly regulated by the cell cycle [34]. DSF or DSF/Cu inhibited cell proliferation, therefore, we further assess whether DSF or DSF/Cu caused cell cycle arrest. The results revealed that DSF or DSF/Cu induced more G0/G1 cell cycle arrest at 36 h than gluconate copper or DMSO in all four cell lines (Fig. 2A–D). 1 μM copper gluconate alone did not change the cell cycle distribution. Our data also demonstrated that the sub-G1 population increased in a time-dependent manner in the cells treated with DSF or DSF/Cu compared with the cells solely treated with gluconate copper or DMSO (Fig. 2A–D). In short, these results suggested that DSF or DSF/Cu caused G0/G1 cell cycle arrest and an increase in the sub-G1 population.

### DSF/Cu induces apoptosis in DLBCL cells

DSF or DSF/Cu markedly decreased cell viability, suggesting that DSF or DSF/Cu may induce cell death in addition to causing cell cycle arrest. Next, it was investigated whether the growth inhibition by DSF or DSF/Cu was caused by apoptosis. Hoechst 33258 and Wright staining revealed that DSF or DSF/Cu-treated DLBCL cells exhibited pyknosis (nucleus condensation) and karyorrhexis (nucleus fragmentation) (Additional file 2: Fig. S2). Thus, the DLBCL cells appeared to undergo apoptosis following DSF or DSF/Cu treatment.

**Fig. 3:**
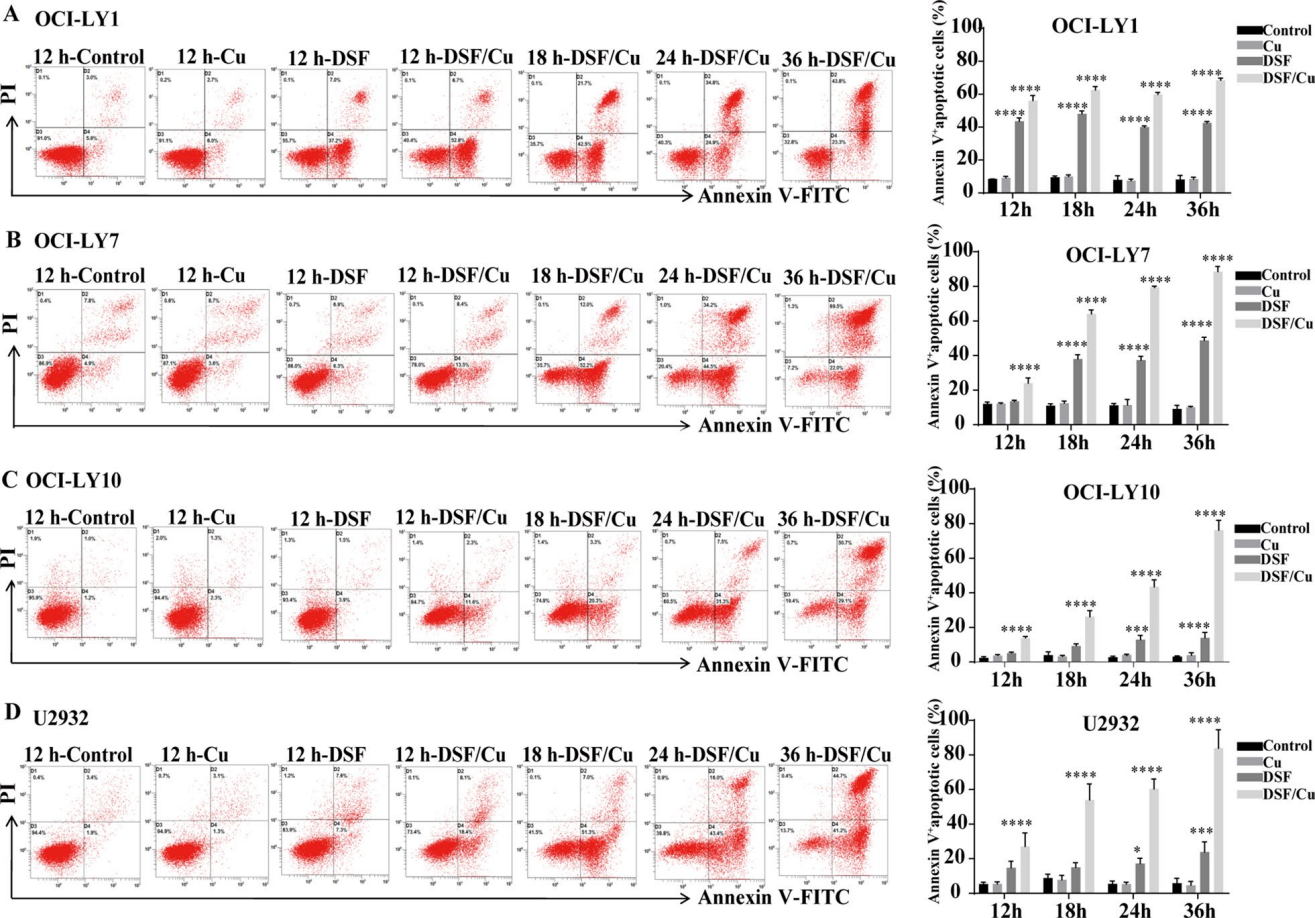
DSF/Cu induces apoptosis in DLBCL cells. DLBCL cells were exposed to DMSO (control), Cu (1 μM), DSF (OCI-LY1: 108.9 nM, OCI-LY7: 104.4 nM, OCI-LY10: 309.7 nM, U2932: 507.9 nM) or DSF/Cu. Annexin V^+^ apoptotic cells were detected using flow cytometry with Annexin V/PI double staining on OCI-LY1 (**A**), OCI-LY7 (**B**), OCI-LY10 (**C**) and U2932 (**D**) cells. Statistical significance were defined at **P < 0.01, ***P < 0.001 and ****P < 0.0001 compared to control

The apoptosis of DLBCL cells was further detected using Annexin V/PI staining by flow cytometry. The results revealed that Annexin V^+^ apoptotic cells were significantly induced by DSF or DSF/Cu in a time and dose dependent manner in all four cell lines. The apoptosis rate induced by DSF/Cu was higher than that induced by DSF alone (Fig. 3A–D). 1 μM copper gluconate alone had no significant effect on the percentage of apoptosis. These findings suggested that DSF/Cu-induced apoptosis is associated with the G0/G1 cell cycle arrest and the increase of the sub-G1 population.

### DSF/Cu decreases mitochondrial membrane potential in DLBCL cells

**Fig. 4:**
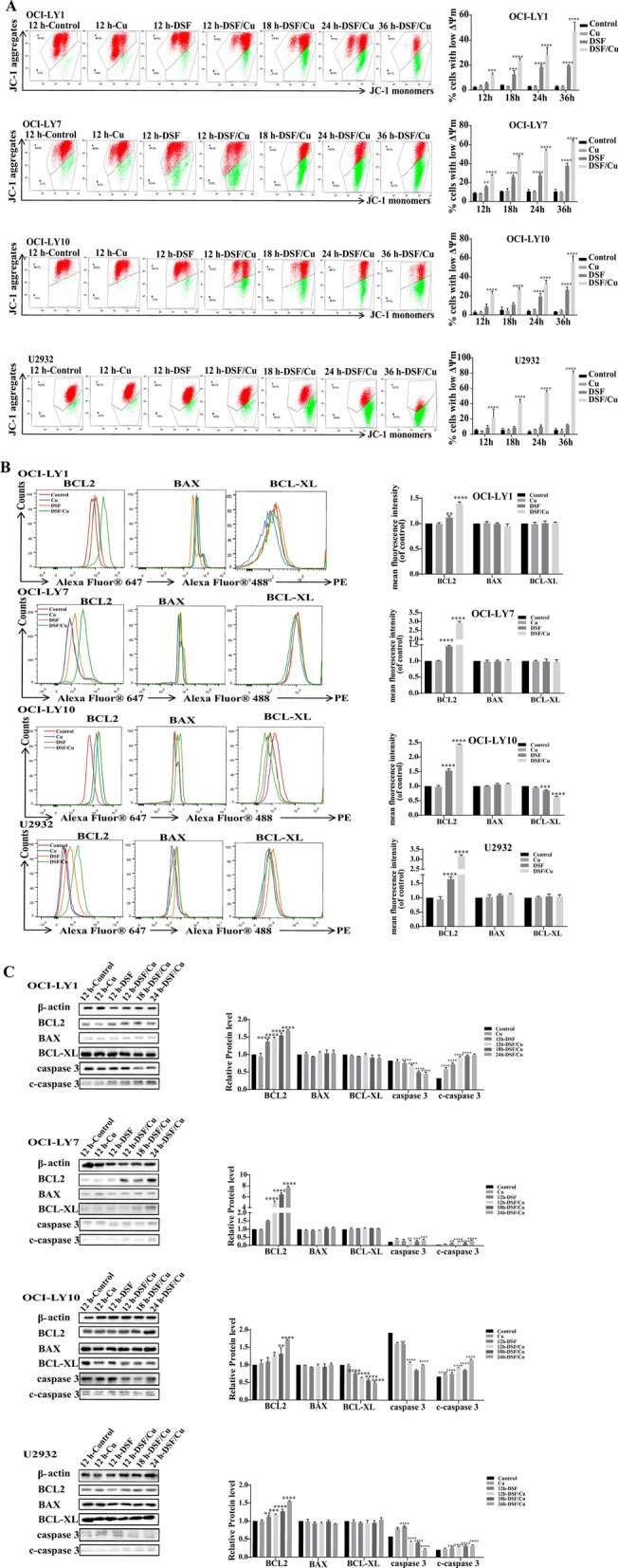
DSF/Cu decreases the mitochondrial membrane potential in DLBCL cells. The mitochondrial membrane potential (**A**) following Cu (1 μM), DSF (OCI-LY1: 108.9 nM, OCI-LY7: 104.4 nM, OCI-LY10: 309.7 nM, U2932: 507.9 nM) or DSF/Cu on OCI-LY1, OCI-LY7, OCI-LY10 and U2932. BCL2, BAX, BCL-XL, caspase 3 and c-caspase 3 in DLBCL cells following Cu, DSF or DSF/Cu at 24 h using flow cytometry (**B**) and western blotting (**C**). Statistical significance were defined at **P < 0.01, ***P < 0.001 and ****P < 0.0001 compared to control

To further reveal the mechanism of DSF or DSF/Cu, the ΔΨm was analyzed using a JC-1 probe by flow cytometry. The results suggested that DSF or DSF/Cu resulted in a significant decrease in the ΔΨm in a time-dependent manner in all four cell lines (Fig. 4A). No obvious difference was observed in DLBCL cells treated with 1 μM copper gluconate alone. These results indicated that DSF/Cu induced ΔΨm loss in DLBCL cells.

The membrane permeability of mitochondria is directly controlled by BCL2 family proteins [35]. At the same time, we detected the expression levels of anti-apoptotic protein BCL2, BCL-XL and pro-apoptotic protein BAX in DLBCL cells following the designated treatments. Surprisingly, we found that the level of BCL2 increased in all four cell lines in a time-dependent manner, while the level of BCL-XL decreased in the OCI-LY10 cell line. BAX levels did not change following the exposure to DSF or DSF/Cu (Fig. 4B, C). Furthermore, Western blot analysis revealed that DSF/Cu induced the expression of c-caspase 3 and promoted caspase 3 cleavage in all four DLBCL cell lines (Fig. 4C). Therefore, we further investigated what induces oncogene addiction switching to BCL2.

### DSF/Cu induce DLBCL apoptosis through decreasing the BCL6

**Fig. 5:**
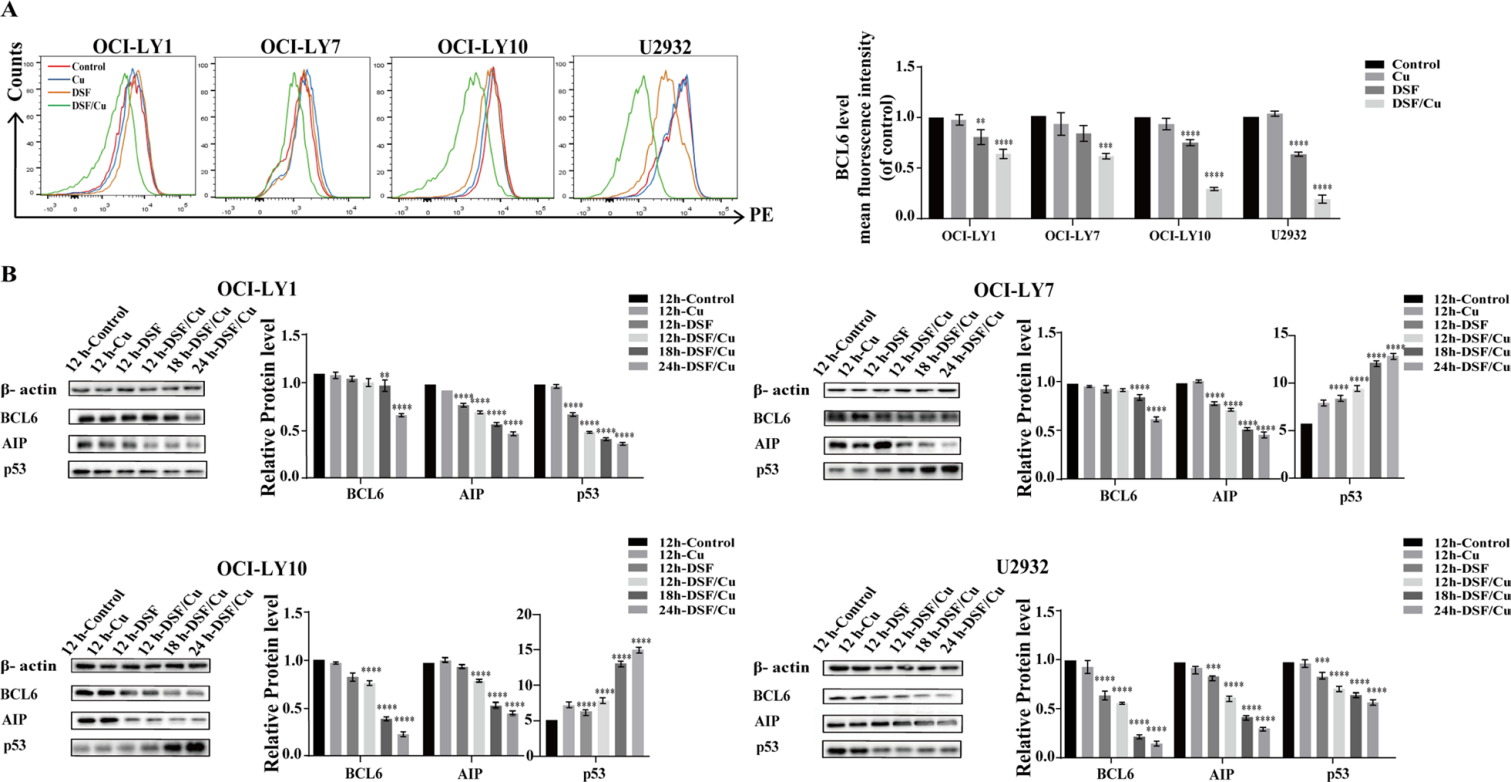
DSF/Cu induce DLBCL apoptosis through decreasing the BCL6. Mean fluorescence intensity of BCL6 and BCL6 level (**A**) following Cu, DSF or DSF/Cu treatment of OCI-LY1, OCI-LY7, OCI-LY10 and U2932 cells. The levels of BCL6, AIP and p53 as well as statistical analysis on OCI-LY1, OCI-LY7, OCI-LY10 and U2932 cells (**B**) following Cu, DSF or DSF/Cu treatment for 24 h. Statistical significance were defined at **P < 0.01, ***P < 0.001 and ****P < 0.0001 compared to control

BCL6 suppresses several prominent B-cell oncogenes including BCL2, BCL-XL and MYC [36, 37]. Therefore, to verify whether the down-regulation of BCL6 may lead to the up-regulation of BCL2, the level of BCL6 in DLBCL cells was next investigated by flow cytometry and western blotting. Interestingly, it was revealed that DSF or DSF/Cu significantly decreased the level of BCL6 in all four cell lines (Fig. 5A, B).

Next, to verify whether DSF/Cu induced DLBCL apoptosis via the BCL6 pathways, we further investigated two BCL6-related protein AIP and p53 through western blotting. As shown in Fig. 5, the level of AIP was down-regulated significantly in all four DLBCL cell lines after DSF or DSF/Cu treatment. Moreover, western blot analysis showed the up-regulation of its downstream targets p53 in OCI-LY7 and OCI-LY10 while the down-regulation of p53 protein in OCI-LY1 and U2932 after DSF or DSF/Cu treatment. This is related to the fact that OCI-LY1 and U2932 expresses mutant p53. Taken together, these data suggest that DSF/Cu may induce DLBCL cell apoptosis via AIP-BCL6-p53 signaling pathway.

### DSF/Cu induces DLBCL cells apoptosis via NF-κB signaling pathways

**Fig. 6:**
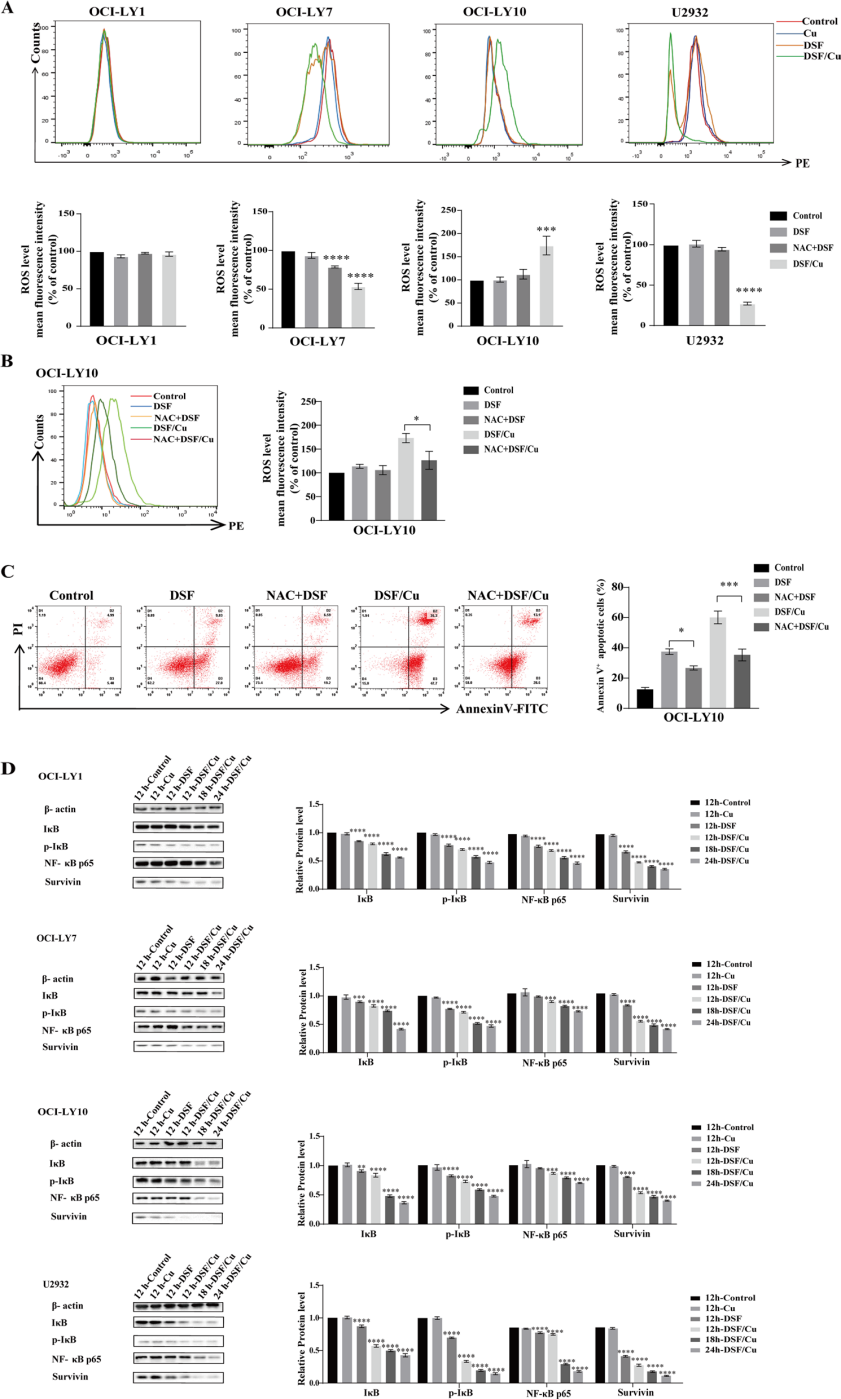
DSF/Cu induces DLBCL cells apoptosis via NF-κB signaling pathways. Mean fluorescence intensity of ROS and ROS level (**A**) following Cu, DSF or DSF/Cu treatment for 24 h of OCI-LY1, OCI-LY7, OCI-LY10 and U2932 cells. ROS level changes (**B**) and annexin V^+^ apoptotic cells (**C**) following DSF, DSF/Cu or NAC + DSF, NAC + DSF/Cu treatment on OCI-LY10. The protein level of IκB, p-IκB, NF-κB p65 and Survivin following Cu, DSF or DSF/Cu on OCI-LY1, OCI-LY7, OCI-LY10 and U2932 cells (**D**). Statistical significance were defined at **P < 0.01, ***P < 0.001 and ****P < 0.0001 compared to control

Next, western blot and flow cytometry were performed to verify whether DSF/Cu induced DLBCL apoptosis via ROS-NF-κB pathways. The intracellular accumulation of ROS was detected using DHR123 fluorescence by flow cytometry. The results demonstrated that DSF or DSF/Cu increased the ROS level of OCI-LY10 cells, but reduced it of OCI-LY7 and U2932 cells (Fig. 6A). As revealed in Fig. 6, 1 μM copper gluconate alone had no significant effect on the ROS levels. ROS in OCI-LY1 cells did not change significantly after DSF or DSF/Cu treatment. Notably, the ROS level and Annexin V^+^ apoptotic cells of OCI-LY10 cells were partly reversed by addition of a ROS scavenger *N*-acetyl-l-cysteine (NAC) (Fig. 6B, C), suggesting that ROS played an important role in DSF-induced apoptosis of OCI-LY10 cells. NAC had no effect on other three cell lines.

Constitutive activation of the NF-κB signaling pathway has been observed in DLBCL [38, 39]. DSF/Cu was reported to induce apoptosis by the alteration of the ROS levels and inhibit NF-κB activities. To determine the effect of DSF or DSF/Cu on the NF-κB signaling pathway, the protein level of IκB, p-IκB, NF-κB p65 and survivin was detected using western blot analysis (Fig. 6D). There was no significant difference in the above protein levels after 1 μM copper gluconate alone treatment. The results revealed that DSF or DSF/Cu inhibited survivin, p-IκB and NF-κB p65 nuclear translocation in DLBCL cells, suggesting that DSF/Cu could induce DLBCL cell apoptosis via NF-κB signaling pathways.

### Antitumor activity of DSF/Cu against primary DLBCL cells

**Fig. 7:**
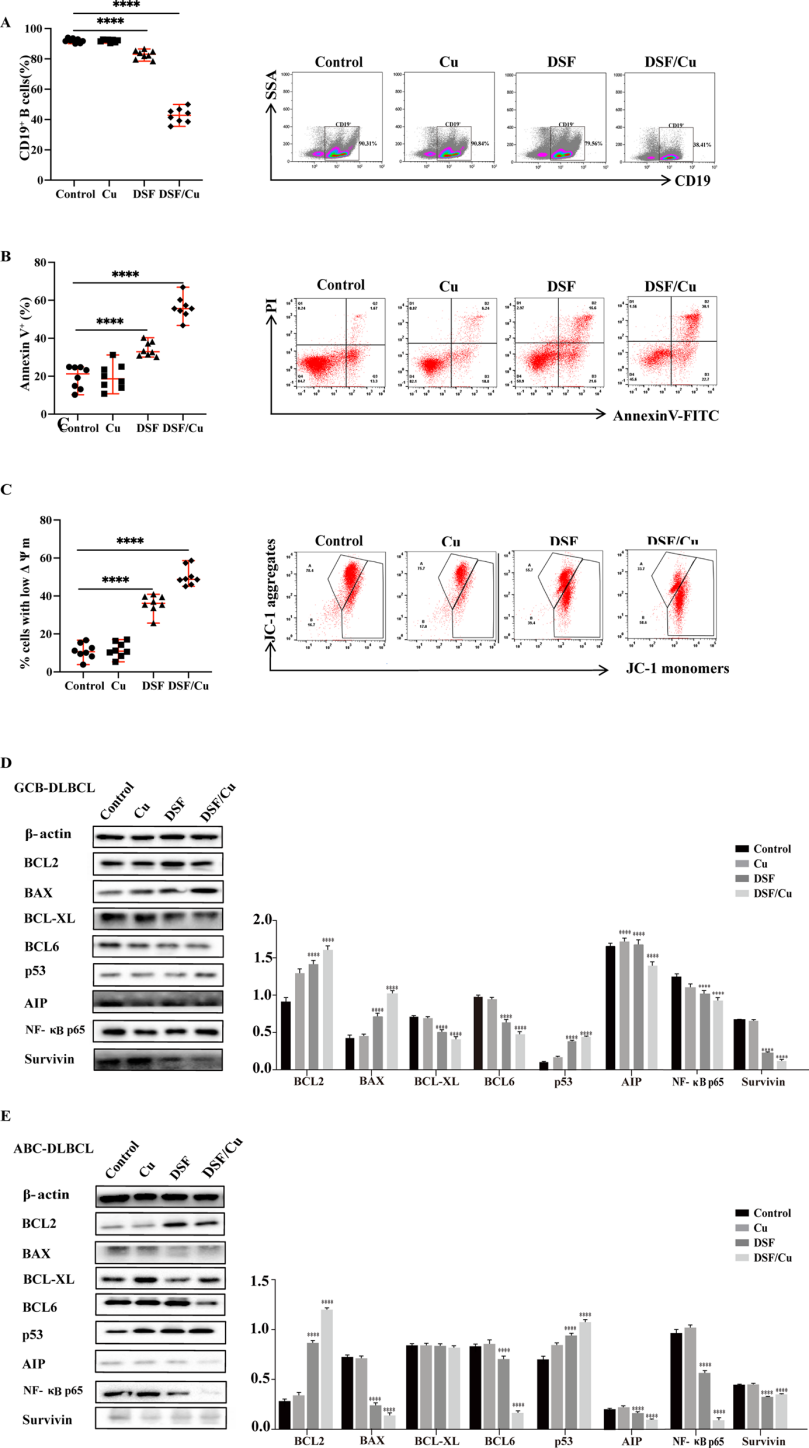
Antitumor effect of DSF/Cu against primary DLBCL cells. Apoptosis statistical chart of primary DLBCL cells (n = 8) and a representative dot plots of a primary DLBCL cells after Cu (1 μM), DSF (400 nM), or DSF/Cu (400 nM/1 μM) treatment for 12 h (**B**). Mitochondrial membrane potential of primary DLBCL cells (n = 8) and a representative dot plots of a primary CD19^+^ B cells from DLBCL patients after Cu (1 μM), DSF (400 nM), or DSF/Cu (400 nM/1 μM) treatment for 12 h (**C**). The protein levels of a primary GCB-DLBCL (**D**) and an ABC-DLBCL (**E**) after DSF or DSF/Cu treatment. Statistical significance were defined at *P < 0.05, **P < 0.01, ***P < 0.001 and ****P < 0.0001 compared to control

To explore the anti-DLBCL activity of DSF/Cu in vivo, we further explored the effects of DSF/Cu on primary DLBCL cells. Primary DLBCL cells were isolated and purified from 3 GCB-DLBCL and 5 ABC-DLBCL patients, and investigate their apoptosis and mitochondrial membrane potential after exposure to DSF (400 nM) or DSF/Cu (400 nM/1 μM) for 12 h. Our data indicated that DSF or DSF/Cu decrease the CD19^+^ B cells and their mitochondrial membrane potential and induced their apoptosis (Fig. 7A–C), suggesting that DSF or DSF/Cu could induce the apoptosis of primary DLBCL cells and may have a therapeutic effect on DLBCL patients. However, the sensitivity of different patients to DSF or DSF/Cu is heterogeneous.

Most interesting, no obvious damage could be observed in CD19^+^ B cells from healthy subjects after DSF or DSF/Cu treatment (Data no shown). Further studies found that, similar to DLBCL cell lines, DSF also induced apoptosis and cyctoxic effect of primary DLBCL cells by inhibiting the NF-κB signaling pathway and down-regulating BCL6 (Fig. 7D, E). In this study, among the 8 DLBCL patients, p53 protein levels were up-regulated in 2 GCB-DLBCL and 1 ABC-DLBCL primary cells, while down-regulated in the remaining 5 primary cells, which indirectly proved that p53 was mutated in the remaining 5 DLBCL primary cells, which was confirmed by our gene detection results.

## Discussion

In the present study, we further confirmed the cytotoxicity effect of DSF on DLBCL cells. Most importantly, the toxic concentration of DSF was lower than the mean plasma concentration of DSF (1.44 μM) [33]. DSF demonstrated a stronger antitumor effect on DLBCL cells in a Cu-dependent manner, which was consistent with previous studies [22, 27, 28, 40]. In addition, DSF or DSF/Cu can induce G0/G1 cell cycle arrest and apoptosis, thereby affecting the proliferation of DLBCL cells.

However, we observed upregulation of the antiapoptotic protein BCL2 in DLBCL cells after DSF or DSF/Cu treatment. BCL6 is known to inhibit the activation of BCL6 target genes such as BCL2, MYC, and NF-κB [41]. Therefore, we further investigated whether BCL6 inhibition leads to oncogene addiction transition to BCL2, and found that the BCL6 level was significantly down-regulated after DSF or DSF/Cu treatment, suggesting that DSF may be a new inhibitor of BCL6.

BCL6 is essential for GC development and plays a major role in controlling B-cell proliferation and differentiation. Our data show that DSF or DSF/Cu induce DLBCL cell apoptosis through down-regulating BCL6, further confirming that BCL6 is necessary for DLBCL cell survival, which is consistent with previous study [37]. Consequently, BCL6 inhibition suppresses lymphoma cells by simultaneously de-repressing multiple genes to deliver powerful anti-proliferation and pro-apoptotic signal to lymphoma cells. The p53 tumor suppressor gene play a role in the tumorigenesis [42]. In normal GC B cells, BCL6 can bind to the p53 promoter region and inhibit p53 transcription, thereby inhibiting apoptosis caused by DNA damage [43]. High expression of BCL6 can immortalize p53-deficient B cells to form lymphoma. AIP also regulates the growth and differentiation of B cells and is structurally expressed in DLBCL. Studies have found that the deletion of AIP in B cells reduces the expression of BCL6 and promotes the germination of GC B cells [17]. In this study, we found the down-regulation of AIP protein level after DSF or DSF/Cu exposure. The p53 protein levels of OCI-LY7 and OCI-LY10 were up-regulated, while the p53 protein levels of OCI-LY1 and U2932 were down-regulated after DSF or DSF/Cu treatment. This is related to the expression of wild-type p53 by OCI-LY7 and OCI-LY10, while mutant p53 by OCI-LY1 and U2932 [44–47]. Our data indicates that DSF/Cu may induce apoptosis via AIP-BCL6-p53 signaling pathways, suggesting that BCL6-related signaling pathways played an important role in DSF/Cu-induced DLBCL apoptosis.

Recently, Bing Xu et al. [24] indicated that DSF/Cu can change cellular ROS levels and inhibit NF-κB signaling pathway in acute myeloid leukemia cells. Consistent with this study, we found that DSF/Cu changed the cellular ROS levels in three cell lines and decreased mitochondrial membrane potential. The dysregulated ROS promote apoptosis mediated by NF-κB signaling pathway [48–50]. Activation of NF-κB in turn inhibits ROS and JNK, ultimately inhibiting ROS-induced apoptosis [51, 52]. NF-κB is frequently expressed in DLBCL and plays a critical role in lymphomagenesis. In our study, NF-κB p65, p-IκB and survivin were down-regulated in all four cell lines after DSF or DSF/Cu treatment. Our results indicate that DSF/Cu trigger DLBCL cell apoptosis via NF-κB signaling pathway.

We further explored the effects of DSF/Cu on primary DLBCL cells, and found that DSF/Cu (400 nM/1 μM) had strong cytotoxic effects on primary DLBCL cells, but had no obvious damage to CD19^+^ B cells in healthy people. Collectively, these data demonstrate that DSF/Cu is safe and specifically targets DLBCL cells in vivo.

## Conclusions

**Fig. 8:**
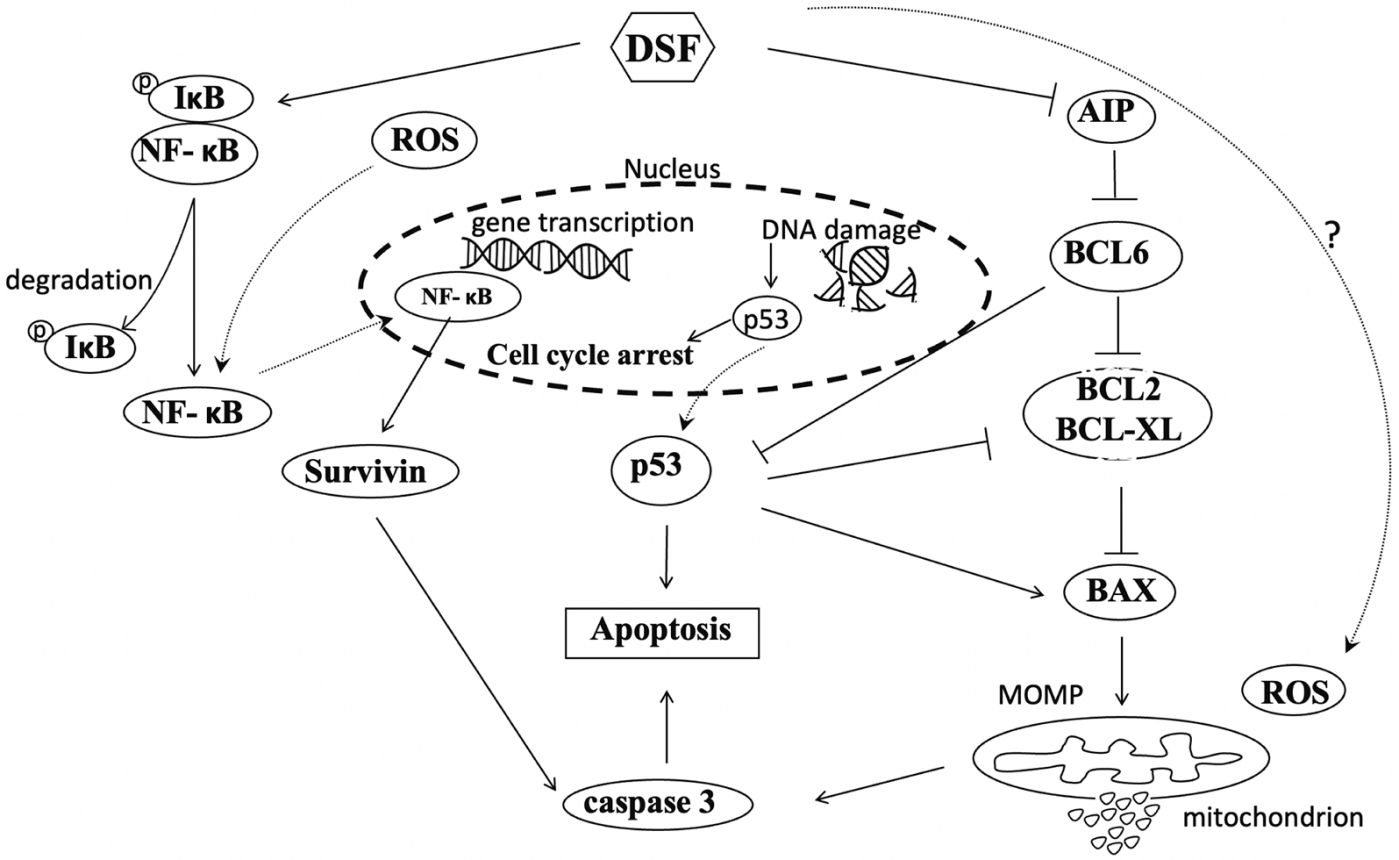
The schematic diagram of DSF/Cu’s molecular mechanism against diffuse large B-cell lymphoma

Taken together, our study revealed that DSF/Cu can strongly induce DLBCL apoptosis in vitro and in vivo. Further investigation revealed that DSF/Cu significantly induced cell apoptosis via inhibitin NF-κB signaling pathway, and down-regulation of BCL6 (Fig. 8). These findings suggested that DSF may be a novel BCL6 inhibitor with potential for the treatment of DLBCL, and further clinical studies are required.

## Supplementary Information

**Additional file 1: Fig. S1.** Microscopic images of DSF or DSF/Cu-treated DLBCL cells. DLBCL cells were exposed to DMSO (control), Cu (1 μM), DSF (OCI-LY1: 108.9 nM, OCI-LY7: 104.4 nM, OCI-LY10: 309.7 nM, U2932: 507.9 nM) or DSF/Cu for 24 h. Cells were viewed by microscopy.

**Additional file 2: Fig. S2.** The Hoechst 33258 staining and the Wright staining of DSF or DSF/Cu-treated DLBCL cells. DLBCL cells were exposed to DMSO (control), Cu (1 μM), DSF (OCI-LY1: 108.9 nM, OCI-LY7: 104.4 nM, OCI-LY10: 309.7 nM, U2932: 507.9 nM) or DSF/Cu for 24 h. Cells were viewed by microscopy after the Hoechst 33258 staining (A) and the Wright staining (B).

## Data Availability

All data generated or analyzed during this study are included in this published article.

## Abbreviations

*BCL6*: The B-cell lymphoma 6
DLBCL: Diffuse large B-cell lymphoma
DSF: Disulfiram
Cu: Copper
ROS: Reactive oxygen species
NHL: Non-Hodgkin lymphoma
ABC: Activated B-cell-like
GCB: Germinal-center B-cell-like
R-CHOP: Rituximab, cyclophosphamide, doxorubicin, vincristine and prednisone
POZ: Pox virus and zinc finger
BTB: Bric a brac
GC: Germinal center
AIP: Aryl Hydrocarbon Receptor Interacting Protein
NF-κB: Nuclear factor kappa beta
CCK-8: Cell Counting Kit-8
IC_50_: 50% Cell growth inhibitory concentration;
MMOP: Mitochondrial outer membrane permeabilization
