# Supplementary figures and images for: DSF/Cu induces antitumor effect against diffuse large B-cell lymphoma through suppressing NF-κB/BCL6 pathways
Source: Cancer Cell Int. 2022 Jul 26;22:236. doi: 10.1186/s12935-022-02661-4 (PMC9317061; doi:10.1186/s12935-022-02661-4)

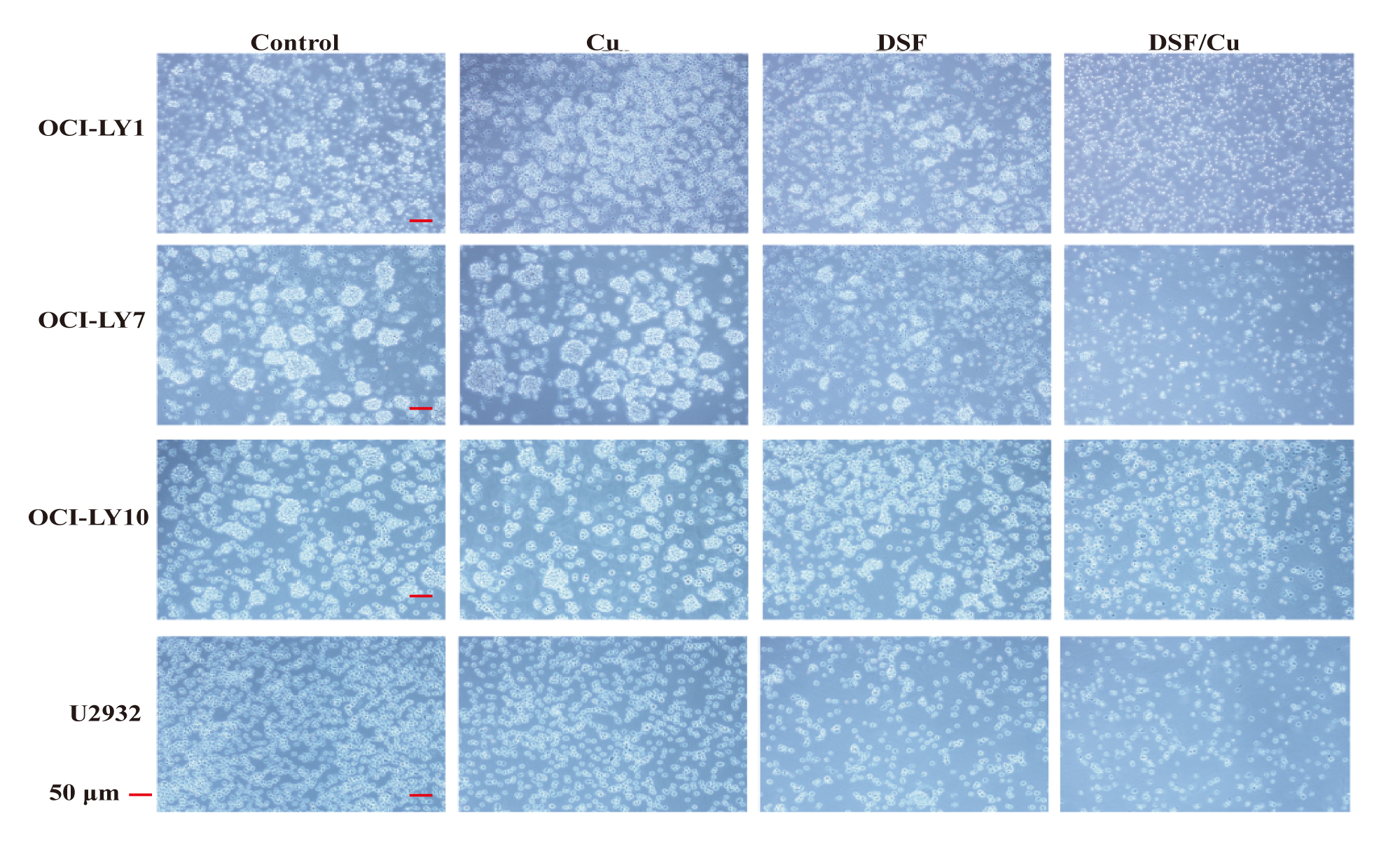

Supplement: Supplementary file 1 — Additional file 1: Fig. S1. Microscopic images of DSF or DSF/Cu-treated DLBCL cells. DLBCL cells were exposed to DMSO (control), Cu (1 μM), DSF (OCI-LY1: 108.9 nM, OCI-LY7: 104.4 nM, OCI-LY10: 309.7 nM, U2932: 507.9 nM) or DSF/Cu for 24 h. Cells were viewed by microscopy. [file 12935_2022_2661_MOESM1_ESM.tif]

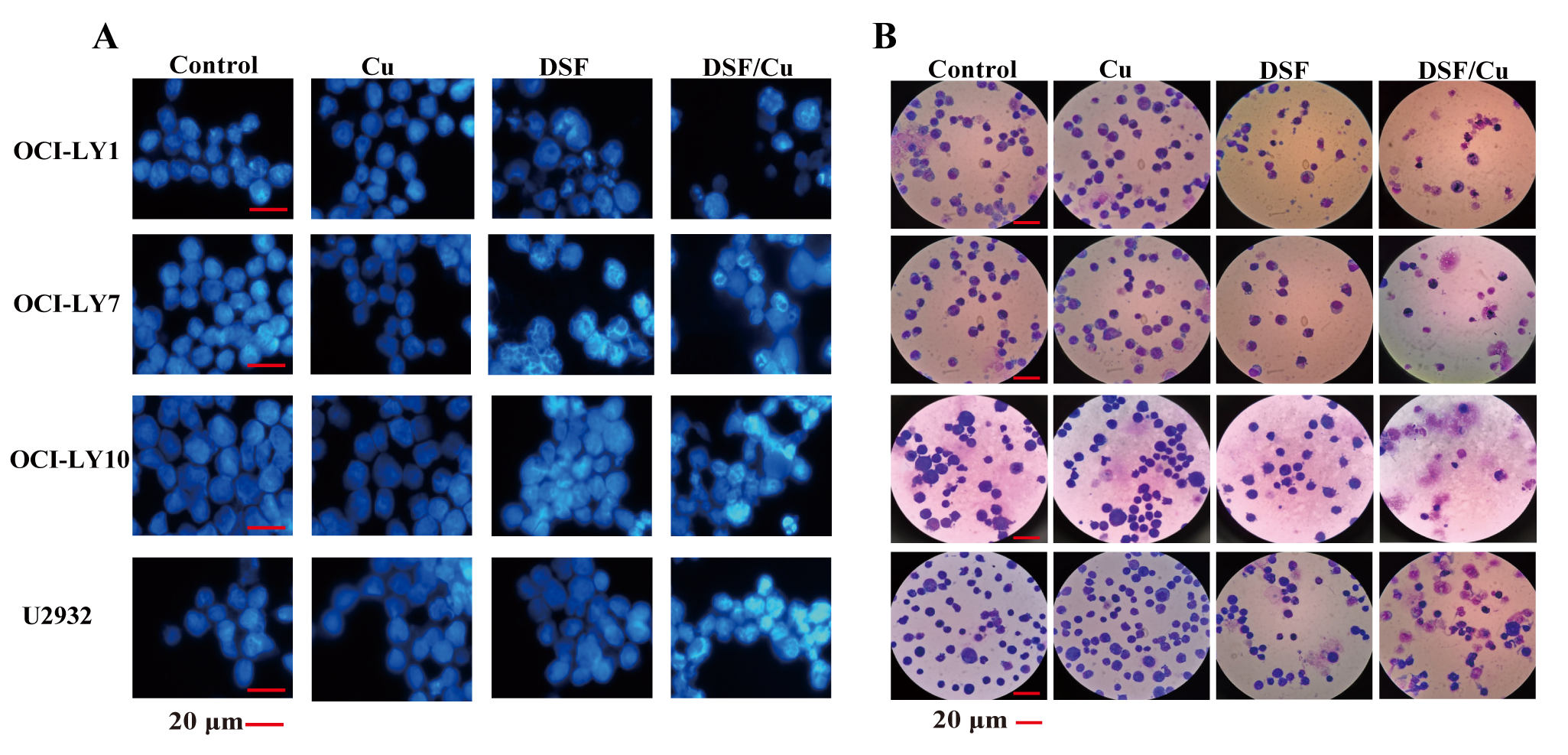

Supplement: Supplementary file 2 — Additional file 2: Fig. S2. The Hoechst 33258 staining and the Wright staining of DSF or DSF/Cu-treated DLBCL cells. DLBCL cells were exposed to DMSO (control), Cu (1 μM), DSF (OCI-LY1: 108.9 nM, OCI-LY7: 104.4 nM, OCI-LY10: 309.7 nM, U2932: 507.9 nM) or DSF/Cu for 24 h. Cells were viewed by microscopy after the Hoechst 33258 staining (A) and the Wright staining (B). [file 12935_2022_2661_MOESM2_ESM.tif]
